# Comments to Article by Solah VA et al., *Nutrients* 2017, *9*, 149

**DOI:** 10.3390/nu9040398

**Published:** 2017-04-18

**Authors:** Vladimir Vuksan, Elena Jovanovski, Andreea Zurbau, Allison Komishon

**Affiliations:** 1 Clinical Nutrition and Risk Factor Modification Centre, St. Michael’s Hospital, Toronto, ON M5B 1W8, Canada; JovanovskiE@smh.ca (E.J.); andreea.zurbau@mail.utoronto.ca (A.Z.); allison.komishon@gmail.com (A.K.); 2 Li Ka Shing Knowledge Institute, St. Michael’s Hospital, Toronto, ON M5B 1W8, Canada; 3 Department of Nutritional Sciences, Faculty of Medicine, University of Toronto, Toronto, ON M5S 1A1, Canada; 4 Department of Medicine, Faculty of Medicine, University of Toronto, Toronto, ON M5S 1A1, Canada; 5 Division of Endocrinology & Metabolism, St. Michael’s Hospital, Toronto, ON M5B 1W8, Canada

Dear Editor,

We read with interest the article published in the 16 February 2017 issue of The Journal, titled “Effect of Fibre Supplementation on Body Weight and Composition, Frequency of Eating and Dietary Choice in Overweight Individuals” by Solah VA et al. [1]. This paper is timely as there is an intensive search for fiber that can affect satiety and promote weight loss. Konjac-based fibers may be viable candidates due to their high viscosity. However, in our opinion there are substantial concerns with regards to the methodology and results as reported in the manuscript.

## Methodological Concerns

This is a three-arm trial which was conducted in 118 overweight individuals randomized to two different forms of PolyGlycopleX^®^ (PGX) fiber blend, receiving 4.5 g gel capsules (*n* = 40), 5 g granules (*n* = 39), or 5 g rice flour control (*n* = 39). 

It is reported that the PGX granules group had a small, but significant, reduction in body weight, waist circumference and frequency of eating occasions by using three statistical approaches: intent-to-treat (ITT), per protocol and a subgroup per “recommended dose” analysis. The authors clearly state that “*for the intention-to-treat analysis, all randomized participants were included*”. However, they subsequently report an ITT analysis which includes 83 subjects instead of the 118 randomized; effectively removing ~30% of study participants, largely from the control group (21 out of 40). The reported within treatment reduction in waist circumference and eating occasions should therefore be interpreted with caution in the absence of an explanation for the exclusion of these subjects or transparent reporting of an a priori ITT model.

The per protocol analysis, which is defined in the manuscript as subjects who completed the 12-week intervention, reports a significant reduction in weight loss and eating occasions, however this is in the subgroup of those who consumed the “*recommended dose*” which includes only 52 subjects (44% of those randomized) not the 83 subjects who completed the intervention. Given the under-powered analysis, these results cannot be interpreted as indicated. It is also unclear how the 52 participants were selected. In Table 5, which reports on the intake of a “*recommended dose*” of three servings per day, does not match the recommendation given in the study design. Compliance data on all subjects who were randomized should be reported, however this is not provided. 

The authors state that the trial used a blinded randomized controlled trial design. It appears that this is not probable as two products were powders administered in foil sachets and one was administered as softgel capsules in a jar and subjects received different instructions depending on the product they received. 

## Reporting

The selected outcome reporting and lack of concordance with trial registration raises another significant caveat. Clinical trial registration lists the frequency of eating occasions as a single primary outcome. This a priori design was changed in the manuscript, which now has five primary outcomes listed: weight, waist circumference, body mass index (BMI), eating occasions and food consumed each day, without defining a single primary outcome. More concerning is that the trial was registered as a two-arm study (PGX flour in sachets and rice flour) that would include 120 participants, but the study was subsequently modified and conducted in three arms and the number of participants remained unchanged. No power analysis is provided. The trial registry includes a multiplicity of secondary outcome measurements such as blood pressure and biochemistry measures (lipids, blood glucose, insulin, insulin sensitivity and apolipoprotein B). None of these measures were reported.

## Conflicts of Interest

The authors declared no conflicts of interest (COI). However, after reading the acknowledgements section, it is apparent that significant COI are present. It is stated that SW received a consultancy fee from InovoBiologic Inc., the manufacturer of PGX; and that RJG is the owner of The Factors Group of Companies, which retains an interest in PGX and has provided financial support. The same authors further failed to disclose patent information where SW is the applicant on two patents on PGX (Publication#: 20150086621 & 20110223192), and RJG is a co-applicant on six patents assigned to InovoBiologic Inc., including four pending (Publication#: 20110223192; 20150086621; 20140120239; 20120040049) and two approved patents on PGX (Publication#: 8062686 and 8597709). In light of declaring no conflict of interest, it is inappropriate that the study sponsor, RJG, “critically reviewed the study design”. 

In summary, ITT analysis results were not reported and conclusions of the study in favor of the intervention are largely overstated as they are based on a selected subgroup of participants. The clinical trial registration does not match the protocol described in the manuscript and COI were not adequately disclosed. Using the actual per protocol analysis results of this study, the correct conclusion should probably be that PGX has little effect on body weight which is in line with the results of a 2015 systematic review [2]. 
